# Supplementary material for: Environment Disaster: A Cross-Sectional Study of the Determinants for the Preparation of Azorean Nurses
Source: Healthcare (Basel). 2025 Feb 2;13(3):303. doi: 10.3390/healthcare13030303 (PMC11817137; doi:10.3390/healthcare13030303)
Supplement: Supplementary file 1 [file healthcare-13-00303-s001.zip › healthcare-3396215-supplementary.pdf]

SUPPLEMENTS

Table S1. Demographic characterization of the sample:

	Features	Absolute frequency	Relative frequency (%)
**Age**	22 to 32 years old	40	17,4
	33 to 43 years old	96	41,7
	44 to 54 years old	67	29,1
	55 to 64 years old	27	11,7
	Total	230	100,0
**Sex**	Male	57	24,8
	Female	173	75,2
	Total	230	100,0
**Marital status**	Married	129	56,1
	Divorced/ Separated	20	8,7
	Single	52	22,6
	Union	27	11,7
	Widowed	2	,9
	Total	230	100,0
**Area of residence**	Rural	77	33,5
	Urban	153	66,5
	Total	230	100,0
**Academic qualifications**	Degree	203	88,3
	Master's Degree	27	11,7
	Total	230	100,0

Table S2. Where you work:

	Absolute frequency	Relative frequency (%)
Hospital of the Autonomous Region of the Azores	134	58,2
Island Health Unit of the Autonomous Region of the Azores	88	38,3
Public-Private Institution	3	1,3
Private Institution	2	,9
Armed Forces	1	,4
Regional Civil Protection and Fire Service of the Azores	2	,9
Total	230	100,0

Table S3. Professional characterization of the sample:

		Absolute frequency	Relative frequency (%)
**Working hours**	Fixed	106	46,1
	Roulement	124	53,9
	Total	230	100,0
	Nurse	135	58,7

**Professional Category**	Specialist Nurse	92	40,0
	Principal Nurse	3	1,3
	Total	230	100,0
**Professional position held**	Head Nurse/Responsible	17	7,4
	Nurse in a Coordination Role	13	5,7
	Specialist Nurse	53	23,0
	General Nurse	147	63,9
	Total	230	100,0
**Professional relationship with the institution**	Fixed-term civil service contract	5	2,2
	Open-ended civil service contract	190	82,6
	Individual employment contract	34	14,8
	Service provider	1	,4
	Total	230	100,0
**Number of hours worked per week**	35 hours per week	187	81,3
	39 hours per week	1	,4
	40 hours per week	40	17,4
	More than 60 hours	1	,4
	Those needed to ensure services	1	,4
	Total	230	100,0
**Area of Specialization in Nursing**	Community Nursing	29	26,4
	Rehabilitation Nursing	14	12,7
	Child and Pediatric Health Nursing	18	16,4
	Maternal and Obstetric Health Nursing	7	6,4
	Mental Health and Psychiatric Nursing	9	8,2
	Medical-Surgical Nursing	33	30,0
	Total	110	100,0
**Workplace in the Autonomous Region of the Azores**	Faial	14	6,1
	Flores	4	1,7
	Graciosa	2	,9
	Pico	5	2,2
	S.º Jorge	3	1,3
	S.º Miguel	164	71,3
	S.ta Maria	6	2,6
	Terceira	32	13,9
	Total	230	100,0
**Island with hospital**	No	20	8,7
	Yes	210	91,3
	Total	230	100,0

Table S4: Descriptive statistics for continuous variables:

	N	Average	Standard Deviation	Median	Range
Years of professional experience	229	18,061	9,184	18,00	1,5-42
Years of professional experience in the current Unit	230	12,572	9,323	11,00	0,25-42

Table S5. Disaster knowledge

	Average	Standard Deviation	Median
7. I am interested in disaster preparedness training that relates specifically to the situation in my community.	5,09	1,15	5,00
18. I know the limits of my knowledge and skills to act as a nurse in disaster situations.	4,92	1,03	5,00
17. I am aware of the potential risks in my community, such as earthquakes, floods, terrorism, etc.	5,20	1,07	5,50
8. I believe that the scientific research literature on disaster preparedness and management is easily accessible.	3,24	1,29	3,00
13. I have a list of contacts in the medical community (community of health professionals) where I work. I have contacts for referral in the event of a disaster (team leader and/or clinical director).	3,36	1,74	3,00
5. I read scientific journal articles on disaster preparedness.	2,39	1,52	2,00
12. I know where to find relevant data or information related to disaster preparedness and management to increase my knowledge in the area.	3,25	1,38	3,00
9. I think the scientific research literature on disaster preparedness is comprehensive.	3,59	1,22	3,50
14. In the event of a disaster, I think there is sufficient support from the local authorities in the municipality, region or at national level.	3,76	1,26	4,00
22. I am familiar with the local emergency response system for disasters.	2,98	1,44	3,00
11. I have difficulty finding relevant information about my community's needs related to disaster preparedness.	3,53	1,35	3,00
3. I know who to contact (emergency and disaster planner) in the event of a disaster in my community.	3,77	1,83	4,00
6. I am aware of the existence of disaster preparedness and management classes, which are offered both at my workplace and at the university or community.	2,57	1,69	2,00
4. I regularly take part in one of the following educational activities: ongoing training, seminars or conferences on disaster preparedness.	2,57	1,66	2,00
2. I participate in drawing up emergency plans and planning for emergency or disaster situations in my community.	1,98	1,59	1,00
15. I participate or have participated in the creation of guidelines and emergency plans that contribute to/influence improvements at local or national level.	1,74	1,44	1,00

Cronbach's alpha=0.864

Table S6. Disaster competence

	Average	Standard Deviation	Median
19. I know how to use personal protective equipment in the event of bioterrorism or biological or chemical attacks.	3,16	1,646	3
23. I am familiar with the triage system used in disaster situations.	3,51	1,797	4
21. I know how to carry out isolation procedures, minimizing community exposure, in the event of bioterrorism, biological or chemical attacks.	2,35	1,371	2
(1) I take part in disaster drills at my workplace (clinic, hospital, etc.) on a regular basis.	2,77	1,835	2
20. I know how to carry out decontamination procedures in the event of bioterrorism	2,17	1,406	2
10. I consider myself prepared for disaster management.	2,59	1,331	2

Cronbach's alpha=0.856

Table S7. Family disaster preparedness

	Average	Standard Deviation	Median
24. I have a personal/family emergency plan in place for disaster situations.	2,58	1,569	2
25. I and my loved ones and family members have agreed how to carry out our personal or family emergency plan(s).	2,28	1,498	2

Cronbach's alpha=0.922

Table S8. Specific knowledge of catastrophe

	Average	Standard Deviation	Median
28. I have knowledge about interventions (psychological, behavioral, cognitive strategies, group support and incident debriefing) to care for people who have experienced emotional or physical trauma.	2,6	1,297	2
29. I am able to describe my role in the response phase to a disaster in my workplace, to the general public, in the media and in personal contacts.	2,95	1,45	3
37. I am familiar with the logistical organization and functioning of local and national entities (operational means of emergency response, e.g. civil protection) in responding to disaster situations.	2,7	1,507	2

Cronbach's alpha=0.776

Table S9. Patient management during the response

	Average	Standard Deviation	Median
39. I feel confident in providing patient training on stress and trauma-related disorders.	2,52	1,407	2
27. I can manage the common symptoms and reactions of disaster survivors of an emotional, behavioral, cognitive and physical nature.	2,98	1,247	3
36. In a disaster situation, I feel confident to take care of people independently, without medical supervision.	2,91	1,504	3
26. I can identify possible indicators of mass exposure evidenced by a group of patients with similar symptoms.	3,43	1,312	4
33. I, as a nurse, feel confident as a manager or coordinator of an emergency shelter.	2,47	1,515	2
34. I, as a nurse, feel confident in my abilities to be a member of a decontamination team.	2,44	1,397	2
32. As a nurse, I feel confident in my abilities to respond quickly as a first responder in a disaster situation.	3,16	1,445	3
38. I feel confident in implementing emergency plans, evacuation procedures and similar functions.	2,67	1,47	2
31. I feel confident in recognizing signs and symptoms indicating potential exposure to biological or chemical agents	2,15	1,196	2
30. I am familiar with the main groups (A, B, C) of biological weapons (anthrax, botulism, etc.), their signs and symptoms and their effective treatment.	1,77	1,15	1
35. In the event of bioterrorism/biological or chemical attack, I know how to draw up a clinical history and assessment specific to the biological and chemical agents used.	1,88	1,119	1

Cronbach's alpha=0.921

Table S10. Knowledge of recovery

	Average	Standard Deviation	Median
42. I am familiar with my role as a nurse in a post-disaster situation	2,93	1,411	3

Table S11. Recovery management

	Average	Standard Deviation	Median
41. I can differentiate between the signs and symptoms of acute stress disorder and post-traumatic stress disorder (PTSD).	2,53	1,363	2
40. I feel confident in providing psycho-education on coping strategies for patients with traumatic experiences so that they can manage them on their own.	2,38	1,345	2
45. I feel confident to act in situations of acute stress disorder or PTSD following an accident or trauma and in follow-ups, taking multidisciplinary (theoretical) references into account.	2,07	1,2	2
44. I am familiar with how to do a health assessment for PPST.	1,91	1,094	2
43. I participate in peer reviews of disaster preparedness and response skills.	2,01	1,325	2

Cronbach's alpha=0.907

Table S12. Comparison of NPC dimensions between professional categories - one-factor ANOVA test:

		n	Average	Standard Deviation	F (gl1, gl2) p-value
Knowledge of disasters	Nurse	135	3,31	0,77	F(2, 227)=1,043 p-value=0.354
	Specialist Nurse	92	3,45	0,89	
	Principal Nurse	3	3,71	1,02	
	Total	230	3,37	0,82	
Disaster competence	Nurse	135	2,62	1,11	F(2, 227)=0,881 p-value=0.416
	Specialist Nurse	92	2,65	1,11	
	Principal Nurse	3	3,48	1,21	
	Total	230	2,64	1,11	
Preparing the family for disaster	Nurse	135	2,39	1,52	F(2, 227)=0,297 p-value=0.743
	Specialist Nurse	92	2,51	1,43	
	Principal Nurse	3	2,00	1,32	
	Total	230	2,43	1,48	
Specific knowledge of catastrophes	Nurse	135	2,74	1,23	F(2, 227)=0,374 p-value=0.689
	Specialist Nurse	92	2,75	1,11	
	Principal Nurse	3	3,33	1,33	
	Total	230	2,75	1,18	
Patient management during the response	Nurse	135	2,58	1,02	F(2, 227)=0,555 p-value=0.575
	Specialist Nurse	92	2,56	0,99	
	Principal Nurse	3	3,18	1,01	
	Total	230	2,58	1,01	

Knowledge about recovery	Nurse	135	2,94	1,43	F(2, 227)=0,467 p-value=0.628
	Specialist Nurse	92	2,88	1,39	
	Principal Nurse	3	3,67	1,53	
	Total	230	2,93	1,41	
Recovery management	Nurse	135	2,18	1,11	F(2, 227)=0,111 p-value=0.895
	Specialist Nurse	92	2,17	1,05	
	Principal Nurse	3	2,47	1,10	
	Total	230	2,18	1,08	

Table S13. Comparison of NPC dimensions between professional roles - one-factor ANOVA test.

		**n**	**Average**	**Standard Deviation**	**F (gl1, gl2) p-value**	**Differences**
Knowledge of disasters	1.Head Nurse/Responsible	17	3,93	0,98	F(3, 226)=9,527 p-value=0.000	1>4* 2>3* 2>4*
	2.Nurse in a coordinating role	13	4,15	0,95		
	3.Specialist Nurse	53	3,45	0,88		
	4 General Nurse	147	3,21	0,70		
	Total	230	3,37	0,82		
Disaster competence	1.Head Nurse/Responsible	17	3,16	0,96	F(3, 226)=3,930 p-value=0.009	2>4*
	2.Nurse in a coordinating role	13	3,41	1,41		
	3.Specialist Nurse	53	2,58	1,20		
	4 General Nurse	147	2,53	1,03		
	Total	230	2,64	1,11		
Preparing the family for disaster	1.Head Nurse/Responsible	17	2,71	1,20	F(3, 226)=2,280 p-value=0.080	
	2.Nurse in a coordinating role	13	2,85	1,70		
	3.Specialist Nurse	53	2,76	1,59		
	4 General Nurse	147	2,24	1,42		
	Total	230	2,43	1,48		
Specific knowledge of catastrophes	1.Head Nurse/Responsible	17	3,22	1,24	F(3, 34)=2,011 p-value=0.131	
	2.Nurse in a coordinating role	13	3,44	1,47		
	3.Specialist Nurse	53	2,67	1,18		
	4 General Nurse	147	2,66	1,13		
	Total	230	2,75	1,18		
Patient management during the response	1.Head Nurse/Responsible	17	3,03	0,86	F(3, 226)=2,436 p-value=0.066	
	2.Nurse in a coordinating role	13	3,06	1,22		
	3.Specialist Nurse	53	2,50	1,09		
	4 General Nurse	147	2,51	0,96		
	Total	230	2,58	1,01		
Knowledge about recovery	1.Head Nurse/Responsible	17	3,53	1,18	F(3, 226)=3,709 p-value=0.012	2>3*
	2.Nurse in a coordinating role	13	3,85	1,63		
	3.Specialist Nurse	53	2,66	1,41		
	4 General Nurse	147	2,87	1,38		
	Total	230	2,93	1,41		
Recovery management	1.Head Nurse/Responsible	17	2,42	,934	F(3, 226)=1,236 p-value=0.297	
	2.Nurse in a coordinating role	13	2,62	1,1		
	3.Specialist Nurse	53	2,06	1,10		

	4 General Nurse	147	2,16	1,08		
	Total	230	2,19	1,08		

Legend: n - number of valid cases (absolute frequency); F - one-factor ANOVA test; gl_1 - degrees of freedom between groups; gl_2 - degrees of freedom between groups; p-value - probability of significance.

*Significant at the 5% level

Table S14. Comparison of NPC dimensions between islands - one-factor ANOVA test.

		N	Average	Standard Deviation	F (gl1, gl2) p-value	Differences
Knowledge of disasters	Faial	14	3,64	0,83	F(7, 222)=4,156 p-value=0.000	Sta. Maria> S.Miguel
	Flores	4	3,63	1,10		
	Graciosa	2	4,56	1,41		
	Pico	5	3,59	0,56		
	S. Jorge	3	3,85	0,75		
	S. Miguel	164	3,23	0,73		
	Sta. Maria	6	4,53	0,88		
	Terceira	32	3,58	0,99		
	Total	230	3,37	0,82		
Disaster competence	Faial	14	2,92	1,03	F(7, 222)=3,907 p-value=0.000	Sta. Maria> S.Miguel
	Flores	4	3,32	1,18		
	Graciosa	2	3,50	1,52		
	Pico	5	2,89	0,84		
	S. Jorge	3	3,05	0,44		
	S. Miguel	164	2,44	1,02		
	Sta. Maria	6	4,12	0,55		
	Terceira	32	3,07	1,38		
	Total	230	2,64	1,11		
Preparing the family for disaster	Faial	14	2,54	1,23	F(7, 222)=3,898 p-value=0.000	S. Jorge> S. Miguel
	Flores	4	2,50	1,22		
	Graciosa	2	2,00	1,41		
	Pico	5	3,00	2,12		
	S. Jorge	3	5,33	1,15		
	S. Miguel	164	2,20	1,37		
	Sta. Maria	6	3,50	2,24		
	Terceira	32	3,03	1,49		
	Total	230	2,43	1,48		
Specific knowledge of catastrophes	Faial	14	2,95	1,08	F(7, 222)=4,087 p-value=0.000	Sta. Maria> S.Miguel
	Flores	4	3,25	1,64		
	Graciosa	2	3,67	2,36		
	Pico	5	3,53	0,77		
	S. Jorge	3	3,56	1,07		
	S. Miguel	164	2,54	1,08		
	Sta. Maria	6	4,44	1,19		
	Terceira	32	3,09	1,30		
	Total	230	2,75	1,18		

Legend: n - number of valid cases (absolute frequency); F - one-factor ANOVA test; gl_1 - degrees of freedom between groups; gl_2 - degrees of freedom between groups; p-value - probability of significance.

*Significant at the 5% level.

Continued:

		n	Average	Standard Deviation	F (gl1, gl2) p-value	Differences*
Patient management during the response	Faial	14	2,69	0,87	F(7, 9)=1,640 p-value=0.238	*No differences*
	Flores	4	3,27	1,16		
	Graciosa	2	2,86	1,61		
	Pico	5	2,91	0,56		
	S. Jorge	3	2,94	0,47		
	S. Miguel	164	2,44	0,97		
	Sta. Maria	6	3,67	1,13		
	Terceira	32	2,87	1,13		
	Total	230	2,58	1,01		
Knowledge about recovery	Faial	14	3,29	1,77	F(7, 9)=2,377 p-value=0.111	*No differences*
	Flores	4	3,50	1,91		
	Graciosa	2	4,50	2,12		
	Pico	5	3,60	0,55		
	S. Jorge	3	3,00	1,00		
	S. Miguel	164	2,71	1,32		
	Sta. Maria	6	4,33	1,21		
	Terceira	32	3,31	1,49		
	Total	230	2,93	1,41		
Recovery management	Faial	14	2,10	0,96	F(7, 222)=4,348 p-value=0.000	Sta. Maria>Faial<br>Sta. Maria>S. Michael<br>Terceira>S. Miguel
	Flores	4	2,50	1,25		
	Graciosa	2	2,90	1,56		
	Pico	5	2,28	0,91		
	S. Jorge	3	2,27	0,90		
	S. Miguel	164	2,00	0,96		
	Sta. Maria	6	3,87	1,04		
	Terceira	32	2,70	1,34		
	Total	230	2,18087	1,083734		

Legend: n - number of valid cases (absolute frequency); F - one-factor ANOVA test; gl_1 - degrees of freedom between groups; gl_2 - degrees of freedom between groups; p-value - probability of significance.

*Significant at the 5% level.

Table S15. Comparison of NPC dimensions between Nursing Specialization Areas - one-factor ANOVA test.

		n	Average	Standard Deviation	F (gl_1 , gl)2 p-value	Differences*
Knowledge of disasters	1. community nursing	29	3,74	0,97	F(5, 104)=3,603 p-value=0.005	1>4<br>6>4
	2. rehabilitation nursing	14	3,19	0,80		
	3. Child and Pediatric Health Nursing	18	3,25	0,72		
	4. Maternal and Obstetric Health Nursing	7	2,66	0,36		
	5. Mental Health and Psychiatric Nursing	9	3,21	0,44		

	6. Medical-Surgical Nursing	33	3,77	0,89		
	Total	110	3,49	0,87		
Disaster competence	1. community nursing	29	2,78	1,17	F(5, 104)=2,834 p-value= 0.019	6>4
	2. rehabilitation nursing	14	2,54	1,31		
	3. Child and Pediatric Health Nursing	18	2,43	0,70		
	4. Maternal and Obstetric Health Nursing	7	1,61	0,53		
	5. Mental Health and Psychiatric Nursing	9	2,51	1,17		
	6. Medical-Surgical Nursing	33	3,15	1,18		
	Total	110	2,71	1,14		
Preparing the family for disaster	1. community nursing	29	2,67	1,45	F(5, 104)=1,531 p-value=0.187	*No differences*
	2. rehabilitation nursing	14	2,36	1,49		
	3. Child and Pediatric Health Nursing	18	2,33	1,08		
	4. Maternal and Obstetric Health Nursing	7	1,43	0,61		
	5. Mental Health and Psychiatric Nursing	9	3,33	1,85		
	6. Medical-Surgical Nursing	33	2,62	1,62		
	Total	110	2,54	1,48		
Specific knowledge of catastrophes	1. community nursing	29	2,87	1,29	F(5, 104)=2,037 p-value=0.079	*No differences*
	2. rehabilitation nursing	14	2,55	1,30		
	3. Child and Pediatric Health Nursing	18	2,63	0,78		
	4. Maternal and Obstetric Health Nursing	7	1,95	0,73		
	5. Mental Health and Psychiatric Nursing	9	3,33	0,88		
	6. Medical-Surgical Nursing	33	3,18	1,25		
	Total	110	2,86	1,18		
Patient management during the response	1. community nursing	29	2,71	1,02	F(5, 104)=1,435 p-value=0.218	*No differences*
	2. rehabilitation nursing	14	2,32	1,17		
	3. Child and Pediatric Health Nursing	18	2,55	0,78		
	4. Maternal and Obstetric Health Nursing	7	1,95	0,64		
	5. Mental Health and Psychiatric Nursing	9	2,65	1,01		
	6. Medical-Surgical Nursing	33	2,94	1,22		
	Total	110	2,65	1,06		
Knowledge about recovery	1. community nursing	29	2,93	1,39	F(5, 104)=2,701 p-value=0.025	5>4
	2. rehabilitation nursing	14	2,57	1,45		
	3. Child and Pediatric Health Nursing	18	3,11	1,02		
	4. Maternal and Obstetric Health Nursing	7	1,86	1,21		
	5. Mental Health and Psychiatric Nursing	9	4,22	1,56		

	6. Medical-Surgical Nursing	33	3,06	1,48		
	Total	110	2,99	1,43		
Recovery management	1. community nursing	29	2,08	1,01	F(5, 104)=3,536 p-value=0.005	5>1 5>3 5>4
	2. rehabilitation nursing	14	2,10	1,19		
	3. Child and Pediatric Health Nursing	18	2,08	0,90		
	4. Maternal and Obstetric Health Nursing	7	1,37	0,48		
	5. Mental Health and Psychiatric Nursing	9	3,44	1,36		
	6. Medical-Surgical Nursing	33	2,33	1,13		
	Total	110	2,22	1,12		

***Table S16*. How I would do to be better prepared (macro category count):**

	Frequency	Valid percentage (%)
Training	138	74,6
Drills/training	24	13,0
Training and drills	14	7,6
Sorting	9	4,9
Total	185	100,0

*Significant at the 5% level.

Table S17. Priority level (macro category count):

	Frequency	Valid percentage
Maximum	98	79,7
Moderate	24	19,5
Low	1	,8
Total	123	100,0

Table S18. Current ability to respond to a variety of disaster situations: (macro category count):

	Absolute frequency	Relative frequency (%)
Low	132	68,8
Moderate	47	24,5
Good	13	6,8
Total	192	100,0

Table S19 (% of respondents who ticked each option)

	n	%
I was taught about catastrophe in my nursing degree.	42	18,3
I was taught about catastrophe in graduate nursing school.	42	18,3
I carried out simulations in facilities as a nurse.	134	58,3
I have attended or am attending disaster courses.	75	32,6
I took part in a disaster (describe what kind of disaster).	28	12,2
I've never been trained in disasters.	7	3,0
I've never taken part in a drill	5	2,2
I acquired training on my own initiative	3	1,3
I got my training from relatives and acquaintances who had lived through disasters	2	0,9
I've carried out simulations in other contexts (secondary education, disaster committees, the service's Internal Emergency Plan, PEE)	3	1,3
I have received training in other contexts	2	0,9
None of the above	12	5,2
I don't remember	1	0,4

Key: n - absolute frequency; % - relative frequency (percentage of respondents who ticked each option)

Table S20 (% of respondents who ticked each option)

	n	%
My role (skills) as a nurse in a disaster situation.	192	83,5
What are the potential risks in my community in the event of a disaster?	140	60,9
Biological and chemical agents and ways of identifying signs and symptoms.	186	80,9
Biological and chemical agents and their differential diagnosis and treatment.	165	71,7
Resources in my community such as referral agencies, health departments, emergency contacts, the chain of command and community shelters.	157	68,3
State of recovery: stress disorder, post-traumatic stress disorder and crisis intervention (focused assessment, briefing strategies and behavioral, cognitive or medication therapy).	155	67,4
I feel prepared to face a catastrophe	34	14,8

Key: n - absolute frequency; % - relative frequency (percentage of respondents who ticked each option)

Table S21. Regularity of drills and their effectiveness

	**Absolute frequency**	**Relative frequency (%)**
No	164	71,3
Yes	66	28,7
Total	230	100,0

Table S22. Authentic experience in disaster situations

	Absolute frequency	Relative frequency (%)
No	32	34,4
Yes	61	65,6
Total	93	100,0

Table S23 Disaster experiences

	Absolute frequency	Relative frequency (%)
No	14	21,5
Yes	51	78,5
Total	65	100,0

Table S24. Preparedness to respond to disasters

	Absolute frequency	Relative frequency (%)
No	197	85,7
Yes	33	14,3
Total	230	100,0
